# The β‐1,3‐glucanosyltransferases (Gels) affect the structure of the rice blast fungal cell wall during appressorium‐mediated plant infection

**DOI:** 10.1111/cmi.12659

**Published:** 2016-10-11

**Authors:** Marketa Samalova, Hugo Mélida, Francisco Vilaplana, Vincent Bulone, Darren M. Soanes, Nicholas J. Talbot, Sarah J. Gurr

**Affiliations:** ^1^ Department of Plant SciencesUniversity of OxfordOxfordUK; ^2^ Division of Glycoscience, School of BiotechnologyRoyal Institute of Technology (KTH)StockholmSweden; ^3^ Centre for Plant Biotechnology and GenomicsUniversidad Politécnica de MadridMadridSpain; ^4^ ARC Centre of Excellence in Plant Cell Walls and School of Agriculture, Food and WineUniversity of AdelaideUrrbraeSouth AustraliaAustralia; ^5^ School of Biosciences, College of Life and Environmental SciencesUniversity of ExeterExeterUK

## Abstract

The fungal wall is pivotal for cell shape and function, and in interfacial protection during host infection and environmental challenge. Here, we provide the first description of the carbohydrate composition and structure of the cell wall of the rice blast fungus *Magnaporthe oryzae*. We focus on the family of glucan elongation proteins (Gels) and characterize five putative β‐1,3‐glucan glucanosyltransferases that each carry the Glycoside Hydrolase 72 signature. We generated targeted deletion mutants of all Gel isoforms, that is, the GH72^+^, which carry a putative carbohydrate‐binding module, and the GH72^−^ Gels, without this motif. We reveal that *M. oryzae* *GH72* ^*+*^ *GEL*s are expressed in spores and during both infective and vegetative growth, but each individual Gel enzymes are dispensable for pathogenicity. Further, we demonstrated that a *Δgel1Δgel3Δgel4* null mutant has a modified cell wall in which 1,3‐glucans have a higher degree of polymerization and are less branched than the wild‐type strain. The mutant showed significant differences in global patterns of gene expression, a hyper‐branching phenotype and no sporulation, and thus was unable to cause rice blast lesions (except via wounded tissues). We conclude that Gel proteins play significant roles in structural modification of the fungal cell wall during appressorium‐mediated plant infection.

## 1 INTRODUCTION

The fungal wall forms a protective barrier against adverse stresses imposed by environmental fluctuations, or during host infection. It acts as a conduit, or harbor, for hydrolytic enzymes or toxins, and is involved in adhesion to abiotic or biotic surfaces. The wall is composed of a reticulate network of stress‐bearing, shape‐conferring polysaccharides with noncovalently and covalently bound embedded proteins, such as glycosylphosphatidylinositol (GPI)‐anchored proteins, and proteins with internal repeats (PIR: Chaffin 2008; Latge 2010). This layered wall carries distinct proportions of β‐glucans (β‐1,3‐glucans, β‐1,6‐glucans, and, in some species, β‐(1,3;1,4)‐glucans (Fontaine et al. 2000), chitin, and proteins, which vary between species, but also with cell type within a given species (Ruiz‐Herrera, Elorza, Valentin, & Sentandreu 2006; Latge 2010; Ruiz‐Herrera & Ortiz‐Castellanos 2010; Mélida, Sain, Stajich, & Bulone 2015). Glucans are the major components of this “generic” fungal wall, dominated by β‐1,3‐glucans. Linear chains of β‐1,3‐glucan are synthesized by a membrane‐localized glucan synthase (Latge 2007; Gastebois et al. 2010a) and are extruded into the wall as polymerization proceeds. Extensive remodeling occurs, most likely in the cell wall, involving formation of β‐1,6 branching points and cross links between β‐glucans and chitin (Aimanianda & Latge 2010; Latge 2010). The orchestration and precise order of the cell wall biosynthetic events and remodeling remains elusive.

Of the various cell wall moieties, β‐1,3‐glucans make up between 40 and 50% of the wall mass *Saccharomyces cerevisiae* and *Candida albicans* (Lipke & Ovalle 1998; Klis, De Groot, & Hellingwerf 2001), and about 60–70% in filamentous fungi such as *Neurospora crassa* (Mélida et al. 2015). In *C. albicans* *,* *S. cerevisiae* *, Schizosaccharomyces pombe*, *Aspergillus fumigatus*, *Fusarium oxysporum, Neurospora crassa,* and *Tuber melanosporum*, the incorporation of nascent β‐1,3‐glucan molecules into the existing β‐glucan network likely involves members of a conserved family, known as the Glycolipid anchored surface proteins (Gas), or Glucan elongation (Gel) proteins (Mühlschlegel & Fonzi 1997; Popolo & Vai 1999; Mouyna et al. 2000a; Caracuel, Martinez‐Rocha, Di Pietro, Madrid, & Roncero 2005; Medina‐Redondo et al. 2010; Kamei et al. 2013; Sillo et al. 2013). Evidence for this comes from *S. cerevisiae* *Δgas1*, which shows a decrease in β‐1,3‐glucan content in the mutant wall, compared with the wild‐type strain, coupled with a rise in β‐1,3‐glucan in the growth medium (Ram et al. 1998). Such data implies that Gas proteins are involved in the incorporation of β‐1,3‐glucan into the wall, but that they are not involved in glucan synthesis (Ram et al. 1998). An analysis of products resulting from *in vitro* incubation of recombinant Gas proteins with a reduced laminarioligosaccharide suggests a two‐step transglycosylating mechanism for these enzymes. Here, Gas proteins cleave a β‐1,3 glycosidic linkage in the glucan chain and subsequently reform a β‐1‐3 linkage between the reducing end of one released chain and the nonreducing end of side branches in existent β‐glucans (Hurtado‐Guerrero et al. 2009). Thus, the transglycosylating activity of Gas proteins leads to the integration of nascent β‐1,3‐glucan chains into the existing ß‐glucan network. However, a role for Gas proteins in incorporating β‐1,3‐glucan into the wall has not been demonstrated *in vivo*. Thus far, the phenotype of *GAS* deletion mutants has been taken as proxy evidence in support of this model, being, specifically, loss of β‐glucan to the medium, reduction in alkali‐insoluble wall glucan, and induction of the cell wall integrity (CWI) pathway (Ram et al. 1998; Fonzi 1999; Carotti et al. 2004; Mouyna et al. 2005; Gastebois, Fontaine, Latge, & Mouyna 2010b).

The filamentous fungus *Magnaporthe oryzae* is the causal agent of rice blast disease (Couch & Kohn 2002). Under blast‐favorable conditions, up to 30% of the annual rice crop can be lost to infection; controlling disease would constitute a major contribution to ensuring global food security (Talbot 2003). Disease is initiated when a three‐celled conidium detaches from conidiophore‐laden host lesions and attaches to the plant surface, by release of apical spore tip mucilage (Hamer, Howard, Chumley, & Valent 1988). Germination leads to formation of a short germ tube, which matures at its tip into an appressorium. This infection structure forms in response to host cues, such as the hard, hydrophobic leaf surface and plant cutin, as well as absence of nutrients (Skamnioti & Gurr 2007; Wilson & Talbot 2009). Autophagy then occurs in the conidium whose content is recycled into the appressorium (Veneault‐Fourrey, Barooah, Egan, Wakley, & Talbot 2006), which is lined with melanin on the inner edge of the fungal wall. Turgor pressure rises within this newly sealed chamber (De Jong, McCormack, Smirnoff, & Talbot 1997), leading to the emergence of a narrow penetration peg, which pushes through the cuticle and cell wall, expands to form a primary hypha, and then differentiates into bulbous invasive hyphae. The fungus spreads rapidly through a susceptible host (Kankanala, Czymmek, & Valent 2007; Khang et al. 2010), culminating in lesions on aerial tissues, which discharge prolific numbers of conidia, thereby promoting epidemic disease spread (Skamnioti & Gurr 2009). The fungus is capable of causing disease on approximately fifty grass and sedge species. Blast disease is thus of concern with regard to its changing demographics and ability to move to new hosts (Yoshida et al. 2016), with its movement fuelled by global climate change (Bebber, Ramotowski, & Gurr 2013).

Our understanding of the mechanisms which underpin pathogenesis remain far from complete, and thus has not yet fuelled the hunt for target‐specific antifungals (Skamnioti & Gurr 2009). Attractive amongst prospective targets is the fungal cell wall. However, little is known about the organization of the *M. oryzae* wall or about wall variation between cell types during plant infection. Previously, research has considered the architecture of the spore surface, revealing a multi‐layered rodlet surface structure, composed of the hydrophobin Mpg1, which is important in appressorium attachment and morphogenesis (Talbot, Ebbole, & Hamer 1993; Talbot et al. 1996; Kershaw, Thornton, Wakley, & Talbot 2005). Electron micrographs by Howard and Valent (1996) and Mares et al. (2006) also showed, respectively, the layered structures of the conidium and hyphal cell, purportedly comprising β‐1,3‐glucans and chitin.

At present, the polysaccharide composition of the *M. oryzae* wall remains unknown. Recently, however, Fujikawa et al. (2009, 2012) revealed that it carries α‐1,3‐glucan moieties and that these surface‐lying polymers play a role in camouflaging the fungus from recognition by the host immune system during formation of infectious hyphae.

In this report, we provide the first detailed profile of the *M. oryzae* wall carbohydrate composition and structure. We consider the roles of the Gel family of β‐1,3‐glucanosyltransferases in infective and vegetative fungal growth. We show that Gel proteins are expressed during infection‐related development and plant infection, and a mutant defective in three Gel enzymes does not cause rice blast disease.

## 2 RESULTS

### 2.1 Putative Gel proteins in M. oryzae

**Figure 1:**
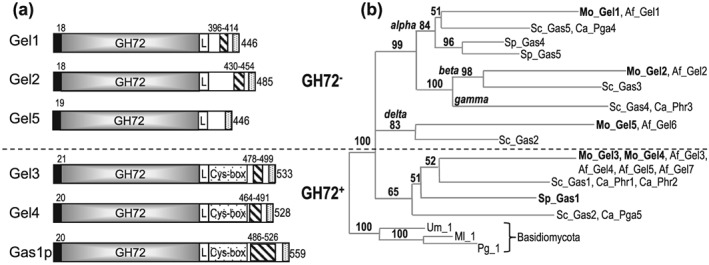
M *agnaporthe* oryzae Gel protein structure and evolutionary phylogenetic tree. (a) Schematic representation of M. oryzae Gel proteins compared to yeast Gas1p. The black and dotted boxes at the N‐ and the C‐terminus are the signal peptide and the GPI‐anchor, respectively. L is the putative linker that links the GH72 catalytic domain (grey) with C‐terminal low complexity region enriched with Ser/Thr (striped box) and the Cys‐box, cysteine‐enriched module, present in GH72^+^subfamily. Note Gas1p contains poly Ser/Thr region unlike any of the *Magnaporth*e Gels. (b) Maximum likelihood phylogenetic tree, comparing Ascomycota Gel proteins of M. oryzae (Mo), A *spergillus* fumigatus (Af), S *accharomyces* cerevisiae (Sc), Candida albicans (Ca) and *Schizosaccharomyces pombe* (Sp) rooted to Basidiomycota Ustilago maydis (Um), *Melampsora larici‐populina* (Ml) and Puccinia graminis (Pg), clearly divides the proteins (indicated by a dash line) into two subfamilies, the GH72^−^cluster (carrying alfa, beta, gamma and delta clades) and the GH72^+^ cluster, which contains the carbohydrate‐binding module of family 43 (Cys‐box). The maximum likelihood tree was adapted from Sillo et al. (2013)

A search of the M. oryzae genome database (http://www.broadinstitute.org) revealed five putative Glucan Elongation (Gel)/Glycolipid Anchored Surface (Gas) proteins, based on sequence similarity to S. cerevisiae Gas1 (Ragni, Fontaine, Gissi, Latge, & Popolo 2007). This family features an N‐terminal signal peptide followed by a catalytic Glycoside Hydrolase 72 domain (GH72) (Pfam: PF03198), a linker region connecting C‐terminal low complexity region with a Ser/Thr percentage of 29–40% (Sillo et al. 2013), and a putative GPI anchor (Figure 1a).

In addition to the GH72 domain, two of these proteins, named Gel3 (MGG_08370.7) and Gel4 (MGG_11861.7), carry a family 43 Carbohydrate Binding Module (CBM43 in CAZy database) also known as an X8 domain (Pfam: PF07983). The CBM43 domain is found in a subset of Gas proteins (Ragni et al. 2007) and carries eight conserved Cys residues (Cys‐box). Based on previous classifications, the two proteins carrying the Cys‐box belong to the GH72^+^ subfamily whilst Gel1 (MGG_07331.7), Gel2 (MGG_06722.7), and Gel5 (MGG_03208.7) belong to the GH72^−^ subfamily (Figure 1a).

To unmask likely evolutionary relationships of *M. oryzae* *GEL* genes, we used maximum likelihood (ML) analysis (Sillo et al. 2013) to compare 237 proteins belonging to 24 Pezizomycotina (e.g*.*, *M. oryzae*, *A. fumigatus*), 25 Saccharomycotina (e.g., *S. cerevisiae*, *C. albicans*), and 2 *Schizosaccharomyces* (*S. pombe*, *S. japonicas*). Three Basidiomycota sequences were used as outgroup taxa (Figure 1b). The tree clearly distinguishes between the GH72^+^ and GH72^−^ subfamilies. Moreover, GH72^−^ could be further divided into alpha, beta, and gamma clades, alongside a newly identified delta clade with members carrying a truncated Cys‐box (6Cys‐box) or no Cys‐box, as with Mo_Gel5. Most Ascomycota carry 3–7 Gel proteins, but Basidiomycota possess only one GH72^+^ *GEL*.

### 2.2 M. oryzae GH72^+^ Gels do not complement yeast *Δgas1*

To investigate Gel3 and/or Gel4 function, we attempted complementation of yeast *Δgas1* mutant. Its phenotype is well characterized; it shows reduced growth, abnormal rounded cells, aberrant budding, increased sensitivity to Congo Red (CR) and Calcofluor White (CFW), oxidative stress, and alkaline pH (Ram, Wolters, Ten Hoopen, & Klis 1994; Ni & Snyder 2001; Serrano, Bernal, Simon, & Arino 2004; Liu, Lee, & Lee 2006; Ando, Nakamura, Murata, Takagi, & Shima 2007). We used the pYES2 heterologous expression system, exploiting the GAL1‐inducible promoter in S. cerevisiae. Mutant cells show reduced growth without induction, when compared with the wild‐type (WT) strain (Figure S1, glucose). However, the addition of galactose restored growth when the original yeast *GAS1* was expressed and cells were plated on galactose‐inducing medium (SG) supplemented with CR, CFW, or SDS. M. oryzae *GEL3* did not complement *Δgas1*; *GEL4* showed partial complementation of *Δgas1* on CFW but not on other growth media. Based on this result, we decided to investigate the M. oryzae GH72^+^subfamily further.

### 2.3 M. oryzae GH72^+^ enzymes are essential for normal vegetative growth under stress conditions

The GH72 domain and Cys‐box of fungal GH72^+^ enzymes have been reported to physically interact and are essential for correct folding and enzyme activity (Popolo et al. 2008; Hurtado‐Guerrero et al. 2009). We thus investigated whether M. oryzae GH72^+^ enzymes play an essential role in wall remodeling by creating single targeted *GEL3* and *GEL4* deletion mutants and a double mutant *Δgel3Δgel4*. To complement the single mutant strains, we fused the *GEL* sequence with a fluorescent protein positioned as an N‐terminal fusion following the signal peptide and expressed the gene under control of its native promoter (Experimental Procedures).

We assayed the effect of various cell wall perturbation chemistries (CR, CFW), applied cell wall and plasma membrane stresses (SDS, alkaline pH, sorbitol, and glycerol), and oxidative stress (hydrogen peroxide). Surprisingly, we observed growth reduction of *Δgel4* and *Δgel3Δgel4* mutants on minimal medium (MM; by approximately 25%), and in CM supplemented with CR (30%) or SDS (25% for *Δgel3Δgel4*; Figure S2). Interestingly, the emergent germ tubes of *Δgel3Δgel4* mutants, germinated in 0.005% (*w*/*v*) SDS, were significantly shorter than Guy11. However, approximately 50% of germlings in the mutant progressed to develop mature appressoria at 24 hpi. The *Δgel3Δgel4* showed reduced growth (by approximately 15%) under oxidative stress, but other factors did not affect growth. The complemented strain *Δgel3/GEL3:mCherry* appeared to be functional but *Δgel4/GEL4:eGFP* only partly restored WT growth.

### 2.4 GH72^+^ *GEL3* and *GEL4* localize to the cell periphery but with different expression patterns in M. oryzae

**Figure 2:**
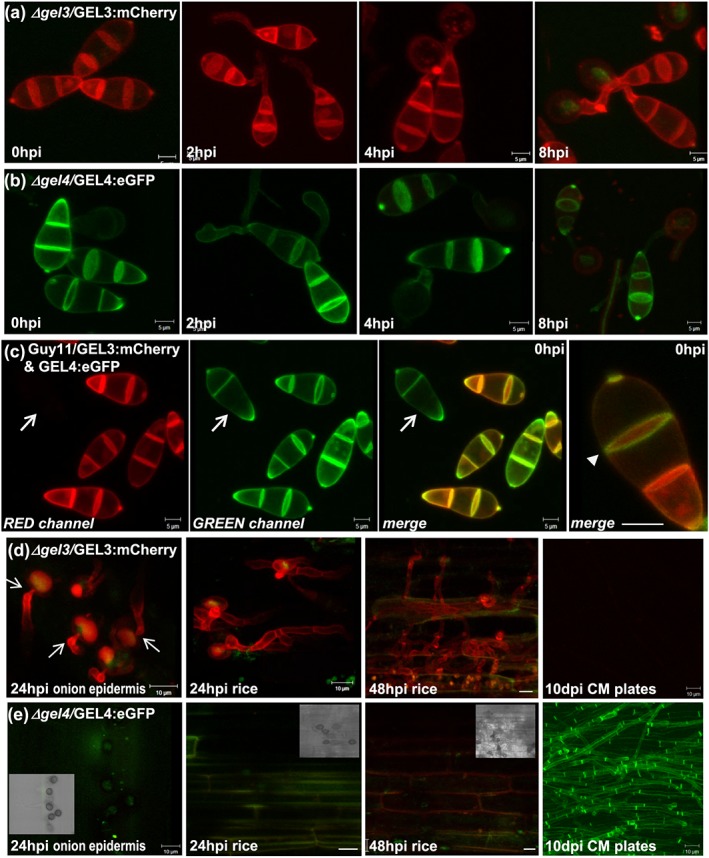
Confocal imaging of fluorescently labeled *GEL3* and *GEL4* at different stages of M *agnaporthe* oryzae development. (a) and (b) Projections of Z‐stacks following spore development on hydrophobic surface (a) *Δgel3* mutant complemented with *GEL3:mCherry* fusion (b) *Δgel4* mutant complemented with *GEL4:eGFP* fusion at 0, 2, 4, and 8 hours post‐inoculation (hpi). (c) Guy11 transformed with both *GEL3:mCherry* and *GEL4:eGFP* fusions at 0 hpi shown in split red and green, as well as merged channels. The arrow points to a spore that is expressing the *GEL4:eGFP* fusion only, therefore appearing invisible in the red channel. The arrow head points to differential circumferential localization of *GEL3:mCherry*, while *GEL4:eGFP* persists along the edges of the spore cell–cell boundaries. Projections of Z‐stacks following expression of (d) *Δgel3*/*GEL3:mCherry* and (e) *Δgel4*/*GEL4:eGFP* during development of penetration pegs (arrows) and infection hyphae on onion peels, at 24 hpi and rice, at 24 and 48 hpi. *GEL4* is not visible at these stages; the transmitted‐light micrograph insert shows that melanized appressoria with invasion hyphae are present. *GEL4* is strongly expressed in vegetative mycelia of 10‐day‐old cultures; *GEL3* is not. The confocal images were collected for both red and green channels to indicate the autofluorescence for the opposite fluorophore. The scale bars are 5 (a, b, c) or 10 (d, e) μm

We used complemented strains *Δgel3/GEL3:mCherry* and *Δgel4/GEL4:eGFP* to localise GH72^+^ *in vivo* by confocal laser scanning microscopy (CLSM), following germling development on hydrophobic glass slides. These surfaces support appressorium differentiation in M. oryzae (Wilson & Talbot 2009). *GEL3* and *GEL4* are both expressed, and their respective protein products localise to the cell periphery of the three‐celled spores and emergent germ tubes up to 4 hours post‐inoculation (hpi; Figure 2a and b). Appressoria were, however, not labeled by the fusions, indeed, by 8 hpi *GEL4* expression is reduced and then disappears completely. When *Δgel3/GEL3:mCherry* and *Δgel4/GEL4:eGFP* fusions were expressed simultaneously in Guy11, some differential labeling was observed; in extreme cases, only *GEL4* was visible but not *GEL3* (Figure 2c, arrow). *GEL3* was more highly expressed and could be tracked during germling development on onion epidermis. This “surface” supports development of penetration pegs and invasive hyphae (Chida & Sisler 1987) (Figure 2d and e). At 24 hpi, the *GEL3:mCherry* fusion highlights a large central vacuole in the appressorium and emerging penetration pegs. Labeling of the cell periphery of invasive hyphae was also clear in infected rice cells, 24 and 48 hpi (Figure 2d). *GEL3:mCherry* was not expressed visibly in vegetative hyphae. By contrast, *GEL4* is not expressed during plant infection but it is expressed in vegetative hyphae (Figure 2). Thus, *GEL3* and *GEL4* are expressed in conidia, but show differential localization during vegetative and invasive hyphal growth, with *GEL3* most strongly associated with host invasion*.*

### 2.5 GH72^+^ Gels are not essential for spore and appressorium development and infection

As *GEL3* and *GEL4* are both expressed during conidial development and *GEL3* is expressed during infection, we investigated the role of GH72^+^ in pathogenicity. We followed germling and appressorium development on hydrophobic glass slides and compared the number of melanized appressoria at 8 hpi between the strains. There was no significant difference between the Guy11, single *Δgel3* and *Δgel4*, and double *Δgel3Δgel4* mutants, or the complemented strains (Figure S2e). Furthermore, we observed no difference in the development of penetration pegs and invasion hyphae on onion epidermis at 24 hpi (Figure S2f). Indeed, the mutants were fully pathogenic on barley (Figure S2g and h).

### 2.6 Monosaccharide composition of M. oryzae cell wall polysaccharides

There has been no detailed analysis of the monosaccharide composition and specific glycosidic linkages of the WT strain Guy11 wall hitherto. We therefore investigated wall monosaccharide composition in Guy11 and compared it with *Δgel3Δgel4*, grown in CM. Total wall polysaccharides were extracted, fully hydrolyzed to their constituent monosaccharides and analyzed by GC/EI‐MS. Table 1 shows only minor differences in total mannose, galactose, glucose and *N*‐acetylglucosamine content between three independent double *Δgel3Δgel4* mutants and Guy11. We also observed that when Guy11 is grown in MM, the wall mannose content was reduced significantly, but was compensated by a significant increase in glucose. Growth conditions thus affect cell wall composition (Aguilar‐Uscanga & Francois 2003).

**Table 1:** Total sugar analysis of the M *agnaporthe* oryzae cell walls (mol%)

	Guy11 MM		Guy11 CM		*Δgel3Δgel4*	
	AV	SEM	AV	SEM	AV	SEM
Mannose	8.5	0.1	15.0	0.1	14.3	0.1
Galactose	0.9	0.0	2.0	0.0	1.6	0.1
Glucose	85.6	0.0	75.7	0.1	77.1	0.1
*N*‐Acetylglucosamine	5.0	0.2	7.3	0.1	7.0	0.2

### 2.7 Targeted deletion of *GEL1, GEL2,* and *GEL5* does not affect fungal development

To investigate the role of the Gel proteins, we created null mutants of GH72^−^ subfamily, *Δgel1*, *Δgel2,* and *Δgel5*. However, no phenotypic differences (germination, germling differentiation assays, or plate growth assays) were observed when various exogenous stresses were imposed (data not shown). Despite protracted efforts, we were unable to visualize GFP or RFP fluorescent tagged GH72^−^ Gels during asexual spore development, penetration and hyphal infection, mycelial growth, or in sexual perithecia and ascospores (data not shown). GH72^−^ *GEL*s appear lowly expressed, as shown by RNAseq data (Soanes, Chakrabarti, Paszkiewicz, Dawe, & Talbot 2012). To confirm this, we used qRT‐PCR to profile expression, revealing only modest fold changes during spore development and early stages of plant infection of all members of M. oryzae *GEL*s (Figure S3a). The most upregulated gene was *GEL2*, which showed a threefold upregulation compared to nongerminated spores at 0 hpi, at 24 hpi, coincident with the time of invasive hypha development. qRT‐PCR results also confirmed that *GEL4* (and *GEL2*) are slightly upregulated in mycelium compared to spores while *GEL3* (and *GEL1*) are downregulated, as seen by confocal microscopy. *GEL5* is weakly expressed in spores but strongly upregulated in mycelium (Figure S3b and S3c).

### 2.8 A Gel‐deficient mutant of M. oryzae is unable to cause rice blast disease

Our observations suggest that GH72^+^ Gel proteins are important in normal mycelial growth under stress conditions. To investigate the coordinated action of the whole Gel family, we introduced deletions in GH72^−^ genes in *Δgel3Δgel4* background to create *Δgel1Δgel3Δgel4*, *Δgel2Δgel3Δgel4,* and *Δgel5Δgel3Δgel4* triple mutants. We also created a *Δgel1Δgel2Δgel5* mutant, thereby deleting all GH72^−^ genes. Finally, we created a double *Δgel2Δgel3* mutant, in which the GH72^+^ and GH72^−^ members, showing elevated expression during spore development and early infection, were deleted.

**Figure 3:**
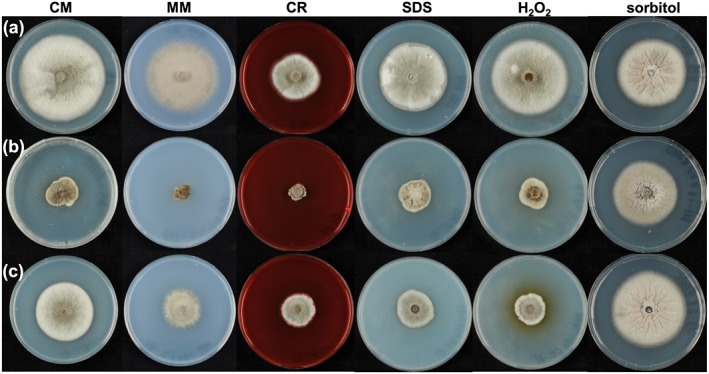
Plate growth assays of wild‐type and triple mutant strains of M *agnaporthe* oryzae. (a) Guy11, (b) *Δgel1Δgel3Δgel4*, and (c) *Δgel2Δgel3Δgel4* strains grown on complete medium (CM), minimal medium (MM), CM supplemented with CR, SDS, H_2_O_2_, and sorbitol at 24°C for 10 days. The experiment was replicated three times with a minimum of two independent lines of each strain; representative pictures are shown

Plate growth assays showed that *Δgel1Δgel3Δgel4*, and to lesser extent, the *Δgel2Δgel3Δgel4*, were hypersensitive to exogenous stresses including plasma membrane and cell wall‐acting agents, as well as to oxidative and heat stress. The treatments included CR, CRW, SDS, NaCl, glycerol, sorbitol, hydrogen peroxide, and elevated temperature (32°C). Most striking was the almost complete inhibition of growth of *Δgel1Δgel3Δgel4* on MM and CM medium supplemented with CR (Figure 3), with growth significantly reduced on CM medium but recovered upon addition of sorbitol or glycerol. The growth of *Δgel5Δgel3Δgel4* was comparable to that of its progenitor strain *Δgel3Δgel4*. *Δgel1Δgel2Δgel5* did not show any growth defects under conditions tested, suggesting that GH72^−^ is dispensable for vegetative growth. Similar results were obtained with *Δgel2Δgel3* (data not shown).

**Figure 4:**
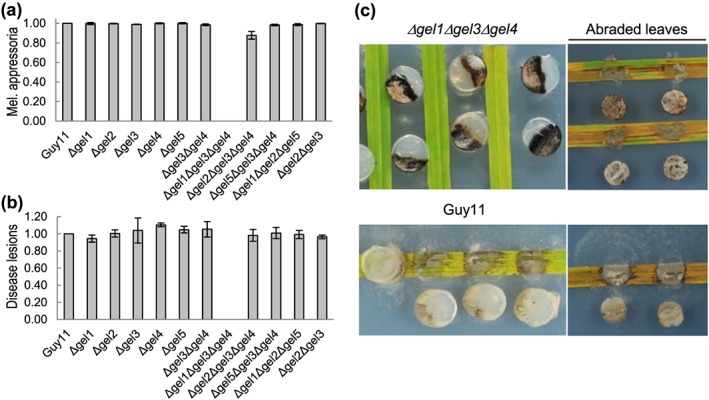
Germling infection‐related development and pathogenicity assays of wild‐type and mutant M *agnaporthe* oryzae strains. Guy11, *Δgel1*, *Δgel2*, *Δgel3*, *Δgel4*, *Δgel5*, *Δgel3Δgel4*, *Δgel1Δgel3Δgel4*, *Δgel2Δgel3Δgel4*, *Δgel5Δgel3Δgel4*, *Δgel1Δgel2Δgel5*, and *Δgel2Δgel3* mutants were assessed for (a) number of melanized appressoria 8 hours post‐inoculation of conidial suspensions (2.5 × 10^−5^ spores ml^−1^) onto a hydrophobic surface. (b) Number of lesions developed on rice (Oryza sativa) leaves spray‐inoculated with conidial suspensions (2.5 × 10^−5^ spores ml^−1^) and incubated for 5 days. Both experiments were replicated three times with a minimum of two independent lines of each strain; results were normalized to Guy11 and shown as mean ± SEM. Note that the triple *Δgel1Δgel3Δgel4* mutant does not produce spores. (c) Representative examples of Guy11 and the *Δgel1Δgel3Δgel4* mutant inoculated as mycelial plugs on rice shown 5 days later when the plugs were removed and placed next to the leaf for photography

Pathogenicity assays of single, double, and triple mutants confirmed that all strains, with the exception of *Δgel1Δgel3Δgel4* (which does not sporulate), produce melanized appressoria (Figure 4a), penetration pegs, and invasive hyphae and are all as pathogenic as Guy11 (Figure 4b). Thus, GH72^+^ and GH72^−^ members are both dispensable for pathogenicity, but specific isoforms are essential for spore formation and host infection (see below).

### 2.9 Δgel1Δgel3Δgel4 has a hyperbranching phenotype and does not produce conidia

*Δgel1Δgel3Δgel4* does not produce fully formed conidia (but occasionally round and terminally swollen hyphal tip cells only), even when plated onto an osmotic medium that supports its growth (Figure 3). Pathogenicity assays were performed with excised and inverted mycelial plugs placed onto a rice leaf. This mode of infection showed that the Guy11 strain causes significant lesion formation at 5 dpi (Figure 4c), but inverted mycelial plugs of the *Δgel1Δgel3Δgel4* mutant do not cause disease symptoms. After leaf cuticle abrasion, however, disease symptoms developed following *Δgel1Δgel3Δgel4* inoculation (Figure 4c), with invasive hyphae invading secondary cells through plasmodesmata.

**Figure 5:**
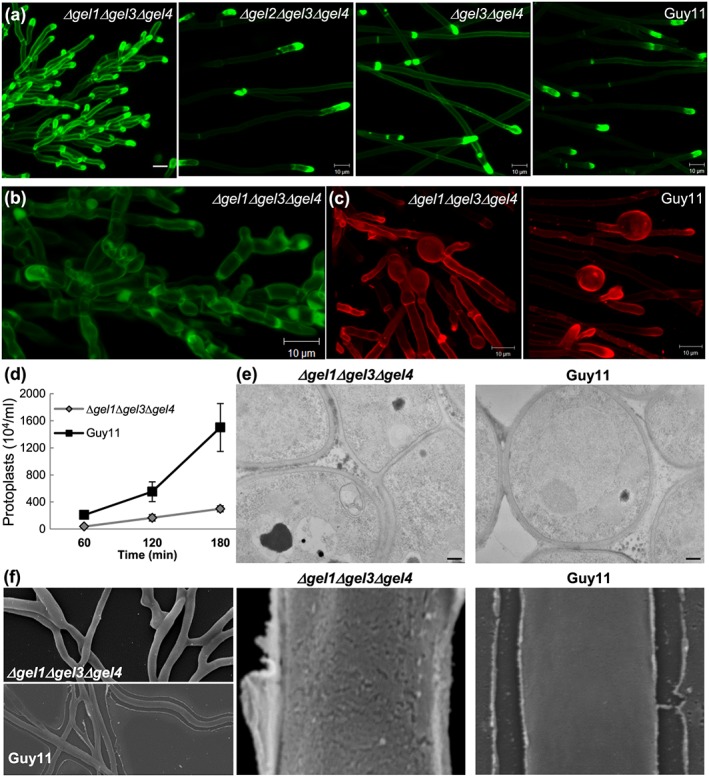
Characterization of triple *Δgel1Δgel3Δgel4* mutant phenotype by confocal microscopy, transmission electron microscopy (TEM), and scanning electron microscopy (SEM). (a) Projection of z‐stack of CFW‐stained growing tips of the triple *Δgel1Δgel3Δgel4* mutant showing hyperbranching phenotype compared to *Δgel2Δgel3Δgel4*, *Δgel3Δgel4*, or wild‐type strain Guy11. The scale bar indicates 10 μm. (b) Higher magnification image of *Δgel1Δgel3Δgel4* mutant stained as in (a) shows very short and rounded cells with multiple branching. (c) Projection of z‐stack of CR stained mycelia near colony edge (5 mm) showing swollen cells in *Δgel1Δgel3Δgel4* mutant that continue to grow, as compared with Guy11 where these are terminal cells. (d) Three‐day‐old Guy11 and *Δgel1Δgel3Δgel4* liquid cultures were exposed to Glucanex for up to 180 minutes post‐treatment and the numbers of protoplast released counted. The experiment was repeated twice with three replica treatments per strain. (e) TEM images of mycelial cross section of *Δgel1Δgel3Δgel4* mutant and Guy11 at 20.000× magnification. The scale bar represents 200 nm. (f) SEM images of *Δgel1Δgel3Δgel4* mutant and Guy11 at 1.700× (left) and 35.000× (middle and right) magnification. The surface of the triple mutant appears rough while Guy11 is smooth but with extruded extracellular matrix

Microscopic observation of the growing edge of *Δgel1Δgel3Δgel4* mycelium also revealed a hyperbranching phenotype (Figure 5a, CFW). In addition, there are differences in general staining intensity, perhaps due to the less branched glucans allowing greater accessibility to CFW, and greater intensity at growing tips, where the newly synthesized glucans are unlikely to have branched or be highly cross‐linked.

The mutant mycelial cells are short, often round, and branch frequently (Figure 5b). Furthermore, when grown across a glass cover slip for 6 days, *Δgel1Δgel3Δgel4* formed terminal rounded tip ends, which then continued to grow and form hyphae (Figure 5c, CR).

Sensitivity to exposure to the fungal wall‐degrading enzyme Glucanex was used to compare the rates of release of protoplasts by *Δgel1Δgel3Δgel4* with Guy11 from mycelial tissues (*Δgel1Δgel3Δgel4* does not sporulate). This revealed that *Δgel1Δgel3Δgel4* releases fewer protoplasts and at a slower rate than the Guy11 strain—approximately 5–10‐fold fewer protoplasts than Guy11, some 180 minutes post‐exposure to wall‐degrading enzymes (Figure 5d). This data suggests that the altered mutant wall is more resistant to Glucanex degradation than WT—a result that attests to the unknown enzyme specificity of these members of the Gel family. *Δgel1Δgel3Δgel4* protoplasts were restored to full growth on CM plates, in a similar manner to Guy11 growth (data not shown).

We compared the mycelial walls of *Δgel1Δgel3Δgel4* and Guy11 by TEM (Figure 5e). This revealed no gross differences in wall thickness between the strains, with *Δgel1Δgel3Δgel4* walls being 81.1 ± 40.6 nm thick and Guy11 walls at 73.8 ± 35.2 nm (*P* = 0.342, *n* = 50)*.* We compared cryo‐SEM images of *Δgel1Δgel3Δgel4* and Guy11 mycelium near its growing edge, showing again the mutant's densely branching phenotype (Figure S4). Finally, we collected SEM images of *Δgel1Δgel3Δgel4* mutant and Guy11, revealing that the mutant surface appears stippled, whilst Guy11 is smooth but with ECM extruded from the wall—a feature absent from the triple mutant (Figure 5f).

### 2.10 Monosaccharide composition and linkage analysis of M. oryzae cell wall polysaccharides in the triple Δgel1Δgel3Δgel4 mutant

**Figure 6:**
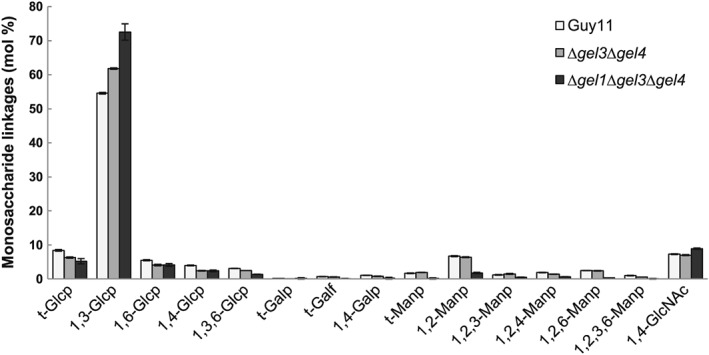
Linkage analysis of purified cell wall polysaccharides from Guy11, *Δgel3Δgel4*, and *Δgel1Δgel3Δgel4* mutant strains (GC/EI‐MS). Liquid complete medium was inoculated with spores or hyphal residues (as the triple mutant does not sporulate) and shaken at 150 rpm at 24°C for 4 or 7 days. Cell wall polysaccharides were purified and analyzed as described in Section 4. The percentage of monosaccharide derivatives identified from each of the three strains was determined from four technical replicates derived from each of the three independently grown biological replicates

We determined the monosaccharide composition of alkali soluble and insoluble fractions (Table 2), and specific glycosidic linkages in the *Δgel1Δgel3Δgel4* wall. Consistent with the double mutant *Δgel3Δgel4,* the triple mutant showed a greater abundance of linear 1,3‐glucans (approximately 18% higher than WT). Indeed, with a decreased proportion in terminal—and 1,3,6‐glycosidic linkages, the glucans are characterized by a higher degree of polymerization and a lower number of 1,6‐branching points (Figure 6). In essence, 1,3‐Glc*p* in *Δgel3Δgel4* (*P* = 0.042, *n* = 3) and *Δgel1Δgel3Δgel4* (*P* = 0.002, *n* = 4), and t‐Glc*p* in *Δgel1Δgel3Δgel4* (*P* = 0.025, *n* = 4); all such values (of double and triple mutant variants) are thus statistically significant from Guy11**.**

**Table 2:** Monosacharide composition of the alkali soluble (ASF) and insoluble (AIF) fraction in M *agnaporthe* oryzae cell walls (mol %)

	ASF				AIF			
	Guy11		*Δgel1Δgel3Δgel4*		Guy11		*Δgel1Δgel3Δgel4*	
	AV	SD	AV	SEM	AV	SD	AV	SEM
Ara	0.74	0.08	7.40	0.08	nd	nd	nd	nd
Xyl	0.13	0.02	2.30	0.14	nd	nd	nd	nd
Mannose	39.80	2.42	30.20	0.68	4.71	1.30	3.55	0.08
Galactose	6.53	0.28	10.93	0.29	1.32	0.37	1.33	0.05
Glucose	51.99	2.50	49.15	0.26	86.53	1.56	86.28	0.45
N‐Acetylglucosamine	0.80	0.15	nd	nd	7.45	0.76	8.90	0.42

### 2.11 Transcriptional analysis of the triple *Δgel1Δgel3Δgel4* mutant strains and Guy11

The triple mutant strain *Δgel1Δgel3Δgel4* shows a nonsporulating, hyper‐branching phenotype. We asked whether this altered morphology correlated with specific changes in genes expression between the mutant and wild‐type strains*—*we thus investigated which genes were differentially expressed as compared with Guy11. We identified global patterns of gene expression in two independent *Δgel1Δgel3Δgel4* mutant strains, compared with Guy11, by RNA‐Seq analysis. Three independent replicates were analyzed from each strain. Figure S5a shows the overall Euclidean distance (distance between two points in space as showing a measure of the differences between the wild type and mutant strains) between all samples. Individual replicates from each sample cluster together and expression data from the two individual mutants are far closer to each other than to Guy11. Based on *p*‐values (adjusted for multiple testing, using Benjamini‐Hochberg method) <0.01 and at least two‐fold difference in expression, the two mutants share 310 genes upregulated and 235 genes downregulated, compared to Guy11 (Table S6 and S7).

GO terms that are more highly represented in genes that showed differential upregulation in *Δgel1Δgel3Δgel4* (as compared with the whole genome) are shown in Figure S5b. Of these, the most interesting are the glycoside hydrolases (GH) (GO:0016798). Nineteen GH encoding genes are upregulated, of which, 14 are predicted to be secreted (Supp Table S8). Fungal cell wall remodeling enzymes include the glucan 1,3‐beta‐glucosidase and chitinase, as well as the wall‐building chitin synthase and polysaccharide‐degrading enzymes, predicted to be extracellular, such as alpha amylase, xylanase, alpha‐galactosidase, and beta‐fructofuranosidase. Interestingly, *GEL2* is upregulated strongly in the mutant, possibly to compensate for the absence *GEL1, GEL3,* and *GEL4*. This follows a similar finding with Gel7 in *Aspergillus fumigatus* (Zhao, Li, Liang, & Sun 2014). The sole gene encoding alpha‐1,3 glucan synthase (*MgAGS1*, Fujikawa et al. 2012) is also upregulated. This gene has been reported to be under the control of MAP kinase Mps1 and therefore may be induced under conditions of cell wall stress (Yoshimi et al. 2013). Other notable differences are shown in Table S9.

GO terms that are more highly represented in downregulated genes found in *Δgel1Δgel3Δgel4* are summarized in Figure S5c and listed in Table S7. They include six genes involved in cell surface signaling (GO:0007166; Kulkarni, Thon, Pan, & Dean 2005), seven genes encoding copper ion‐binding proteins (GO:0005507)—two of which are involved in conidial pigment biosynthesis (Figure S7), five chitin‐binding proteins (GO:0008061), and also *MGG_02246*, a homologue of N. crassa highly expressed conidiation‐specific protein 6 (White & Yanofsky 1993)*.* Both *GEL1* and *GEL4* are significantly downregulated in *Δgel1Δgel3Δgel4*.

Thus, many of the changes in gene expression identified in *Δgel1Δgel3Δgel4* are likely due either directly or indirectly (because of exogenously imposed wall stress), to the lessened proportion of wall ß‐1,3‐glucans. The elevated expression of a number of secreted proteases and certain wall‐remodeling enzymes may also be in response to changes in wall composition. Indeed, in C. albicans, secreted protease activity influences wall function, by proteolytic cleavage of wall proteins (Schild et al. 2011). We conclude that the Gel‐deficient *Δgel1Δgel3Δgel4* mutant shows significant differences in gene expression of a wide range of wall‐encoding enzymes. This highlights the global effect of perturbation of the β‐1,3‐glucan content and the impact this structural modification has on cell wall composition and fungal virulence.

## 3 DISCUSSION

During plant infection, the rice blast fungus undergoes a series of morphogenetic transitions. These include development of the appressorium and formation of invasive hyphae that colonize rice cells and propagate by pseudohyphal growth, a feature not observed in vegetative culture. In this report, we provide the first comprehensive description of the wall composition in the rice blast fungus, which is related to the developmental biology of the pathogen. In *M. oryzae,* glucosyl residues dominate, representing 75% of the monosaccharide components of the wall. The other monosaccharides occurring in the fungal wall are mannose (14%), *N*‐acetylglucosamine (7%), galactose (2%), and traces of arabinose and xylose. We are aware of only one other plant‐pathogenic fungus where the wall has been described in detail, that is, the necrotroph *Botrytis cinerea* (Cantu, Greve, Labavitch, & Powell 2009). The glucose component appears higher in the *B. cinerea* wall, with approximately 90% glucose and much lower amounts of galactose, mannose, and arabinose (Cantu et al. 2009). The *M. oryzae* data align well with the wall compositions described in *S. cerevisiae* (Dallies, Francois, & Paquet 1998) and C. albicans (Ene et al. 2012), whereas in A. fumigatus and *S. pombe,* galactomannans are more prevalent, but such analyses account for both the wall and ECM (Xie & Lipke 2010). It is important to note that the amounts of each the constituent monosaccharides are not absolutes as they fluctuate during growth and morphogenesis, and in response to external stress or medium composition.

As the fungal wall comprises components unique to the Kingdom Fungi, it forms an attractive target for the development of novel antifungal drugs. Indeed, towards the goal of rational design of novel antifungals, it is prescient to characterize the proteins considered to catalyze early steps in the formation of the uniquely fungal elongate and branched chains—that is the step, likely driven by Gel activity in fungi. These proteins are tethered to the plasma membrane by GPI anchors and face the wall: They are thus perfectly placed to create branch points/branches on the main backbone of the emergent chain.

We thus investigated the GH72 family of putative β‐1,3‐glucanosytransferases. In *M. oryzae*, 5 *GEL* genes encode the family GH72^+^ (*GEL3* and *GEL4*), the GH72^−^ (*GEL1*, *GEL2,* and *GEL5*). These proteins have been investigated in a number of fungal species, including *S. cerevisiae* (Ragni et al. 2007), *S. pombe* (Medina‐Redondo et al. 2010), and the filamentous fungi *C. albicans* and *A. fumigatus* (Mouyna et al. 2000b; Mouyna et al. 2005). The Gels display β‐1,3‐glucanosyltransferase activity *in vitro*, although they differ in their specificity for substrate length, cleavage point, and product size. However, when we overexpressed *M. oryzae* *GEL3* and *GEL4* in yeast *Δgas1,* neither fully complemented the mutant phenotype (despite having both functional GH72 and CBM43 domains) suggesting a different role for these proteins in the rice blast fungus. Despite protracted effort, we were unable to express *GEL4* in heterologous expression systems in *P. pastoris* and *E. coli*. Gel3 was successfully expressed, albeit at very low levels, but its instability precluded *in vitro* enzymatic assays (data not shown). Nevertheless, detailed linkage analysis of the wall polysaccharides of *Δgel3Δgel4* revealed increased proportions of 1,3‐linked glucose residues, while the proportions of terminal glucose and residues indicative of the presence of branching points (1,3,6‐Glc*p*) were less abundant. These data suggest that the proteins function on the 1,3‐glucan chains and might be involved in branching activity indirectly.

We localized Gel3 and Gel4 to the cell wall periphery by creating internal fusions with mCherry and GFP, and expressing them under their respective native promoters. Similar localization was reported for YFP‐gas1 and gas2‐GFP fusions in *S. pombe* (Medina‐Redondo et al. 2010) and Phr1‐GFP fusion in C. albicans (Ragni et al. 2011). We showed spatial and temporal differences in expression between the two genes: Both are expressed in ungerminated and germinated spores, and germ tubes but do not completely co‐localise. This was demonstrated in the WT strain transformed with both fusions: For example, while the Gel4‐GFP localized more to the periphery of the conidial septum between the basal and middle cell, the Gel3‐mCherry was uniformly dispersed within the septum. Similar observations were made in *S. pombe* where Gas1p localized as a disc to the nascent septum, whereas Gas2p remained at the septum edging during its synthesis (Medina‐Redondo et al. 2010).

Single *GEL* gene deletions of all family members did not reveal any phenotypic differences from Guy11, apart from reduced growth of *Δgel4* on MM or on CM supplemented with CFW or SDS, as reported for many CW mutants (Maddi, Dettman, Fu, Seiler, & Free 2012). This finding resonates with the observation that *GEL4* is strongly expressed in mycelium. This phenotype was further enhanced in double *Δgel3Δgel4* mutant, which was also sensitive to oxidative stress. However, both GH72^+^ gene mutant strains, (*Δgel3Δgel4*), as well as GH72^−^ mutant, tested as *Δgel1Δgel2Δgel5* proved dispensable for pathogenicity.

From the combinatorial triple deletion strains generated in this study, *Δgel1Δgel3Δgel4* is a non‐sporulating hyperbranching mutant emanating from shortened hyphal cells. The mutant does not infect intact rice leaves but it can cause lesion formation when mycelium is inoculated onto an abraded cuticle. Detailed analysis of this triple mutant strain reveals that it is more resistant to digestion by glucan‐degrading enzymes than WT, as has been demonstrated previously in *N. crassa* (Kamei et al. 2013). There is, however, no obvious difference in cell wall thickness, as evidenced by TEM. The mutant strain wall appeared rougher than the WT wall, and ECM was absent from *Δgel1Δgel3Δgel4*.

Cell wall analysis revealed only minor differences in the glucose between the *Δgel1Δgel3Δgel4* mutant and WT, but galactose is significantly increased, while mannose reduced. Perhaps the most surprising is the 10‐fold increase in arabinose and xylose in the triple mutant. However, these are minor components of the mutant wall suggestive of the presence of arabinoxylans. The linkage analysis further confirmed the observation made with the double *Δgel3Δgel4* mutant, that is, an increased number of 1,3‐glucose linkages in the triple mutant strain.

When considered together, we have invoked the use of *GEL* gene deletions to show that the cell wall composition of *M. oryzae* differs during infection‐related development, and we have described the differential contributions of the family of ß‐1,3‐glucan glucanosyltransferases. These enzymes play key roles in the development and structural composition of conidia and germ tubes, but do not contribute to the rigid cell wall associated with the melanin‐pigmented appressorium that is formed by the fungus to bring about plant infection. Although individually dispensable for virulence of *M. oryzae*, a mutant lacking three of the *GEL*‐encoding genes, *Δgel1Δgel3Δgel4,* was unable to cause rice blast disease and also showed a different developmental phenotype, with a hyper‐branching hyphal phenotype and the absence of spores. This suggests that the structural integrity and flexibility of the cell wall is adversely affected by the disruption to ß‐1,3‐glucan glucanosyltransferase activity. This also, however, clearly has wider impacts, based on RNA‐seq analysis, which revealed an effect not only on perturbed expression of genes encoding cell wall‐associated enzymes, but on many membrane proteins associated with surface sensing, such as G‐protein‐coupled receptors. Taken together, this highlights the interplay and reliance of membrane signaling on the structural properties of the fungal wall, and how perturbation of wall characteristics can exert a profound effect on external communication by fungal cells, which affects their ability to undergo the developmental transitions required for host infection.

## 4 EXPERIMENTAL PROCEDURES

### 4.1 Fungal strains and growth conditions

The WT rice pathogenic *M. oryzae* strain Guy11 and mutants were cultured at 24°C, with a 14‐h light 10‐h dark cycle. Strain maintenance and media composition are as Talbot et al. (1993).

### 4.2 Targeted deletion of *M. oryzae* *GELs*

To generate single targeted gene deletions *Δgel1*, *Δgel2*, *Δgel3*, *Δgel4*, *Δgel5*, *M. oryzae* *GEL1* (*MGG_07331*) and *GEL3* (*MGG_08370*) were replaced by a hygromycin resistance cassette (Sweigard, Chumly, Carrol, Farrall, & Valent 1997); and *GEL2* (*MGG_06722*), *GEL4* (*MGG_11861*), and *GEL5* (*MGG_03208*) by the bialophos resistance marker (GenBank AF013602). Fragments carrying approximately 1.5 kb upstream and 1.2 kb downstream of GEL‐specific flanking sequences were PCR amplified using primers GELx‐KO‐F + pGELx‐R and pAGELx‐F + GELx‐KO‐R, respectively. Fragments were conjoined, amplified using pGELx‐F + pAGELx‐R primers, and carrying a selectable marker, by over‐lapping PCR using GELx‐KO‐F + GELx‐KO‐R primers and the amplicon used directly for DNA‐mediated protoplast transformation of Guy11 (Talbot et al. (1993). Putative transformants were selected on MM supplemented with 300 μg/ml^−1^ hygromycin B (Calbiochem, Merck, Darmstadt, Germany) or defined complex medium (DCM) supplemented with 60 μg/ml^−1^ Bialophos (Goldbio, St Louis, MO, USA); subjected to PCR and Southern blot analysis to confirm single targeted gene replacement, as in Samalova et al. (2013) (Figure S6). To generate double knock‐outs Δ*gel3*Δ*gel4*, Δ*gel3* was retransformed with *GEL4;* Δ*gel2*Δ*gel3*, Δ*gel3* was retransformed with *GEL2;* Δ*gel1*Δ*gel5*, Δ*gel5* was retransformed with *GEL1*.

To generate triple knock‐outs *Δgel1Δgel3Δgel4*, *Δgel2Δgel3Δgel4, Δgel5Δgel3Δgel4*; *Δgel3Δgel4* was retransformed with *GEL1*, *GEL2,* or *GEL5,* respectively. Finally, to generate *Δgel1Δgel2Δgel5;* Δ*gel1*Δ*gel5* was retransformed with *GEL2*. We used a third selectable marker, a resistant allele of M. oryzae *ILV1* gene (*MGG_06868*) to sulphonylurea in pCB1532 plasmid and GAP‐repair S. cerevisiae cloning (Oldenburg, Vo, Michaelis, & Paddon 1997), to assemble the constructs in pNEB1284 (primers detailed in Table S3). Putative transformants were selected on BDCM medium, supplemented with 100 μg/ml^−1^ chlorimuron ethyl (Sigma Aldrich, UK), and confirmed, as above.

### 4.3 Confocal imaging

Spores (2.5 × 10^5^/ml^−1^) of Guy11 and complemented strains were collected from 10‐day old plates and inoculated in 50‐μl droplets onto hydrophobic glass cover slips (0, 2, 4, 8, and 16 hpi), onion peels (24 and 48 hpi), or rice leaf sheaths (24 and 48 hpi), as in Samalova, Meyer, Gurr, and Fricker (2014). To image vegetative hyphae, a glass cover slip was coated with a thin layer of growth medium; placed by the fungal growing edge and left to overgrow for two days. The coverslip was lifted off and the edge imaged using the C‐Apochromat 40×/1.2 water‐corrected objective lens of a Zeiss LSM510 Meta confocal microscope at 500–530 nm, with 488‐nm Argon laser for eGFP, and 543‐nm HeNe laser and BP565–615 filter for mCherry.

The CFW and CR staining was performed by overgrowing Guy11 and mutants on cover slips for 2–6 days then a drop of CFW or CR, at concentration 0.5 mg/ml^−1^, was added 1 hr prior to imaging. The samples were viewed using CLS microscope, with 405‐nm excitation and LP420 filter for CFW, and 543‐nm excitation and LP585 for CR.

### 4.4 Plate growth assays

Radial colony growth was assessed on CM and MM. CM plates, supplemented with CR: 150 mg/L^−1^, CFW: 40 mg/L^−1^, 0.005% (*w*/*v*) SDS, 0.5 M NaCl, 1 M sorbitol, 1 M glycerol, and 5 mM H_2_O_2_, were inoculated with a mycelial plug of 10‐day‐old plates (or 21‐day‐old plates for *Δgel1Δgel3Δgel4* mutant) and incubated for 10 days at 24°C in dark; apart from CM, MM, and SDS plates, grown under normal light cycle conditions. Heat stress was by moving CM plates to 32°C, 3 days post‐inoculation. Colony diameters were measured; assays were with minimum four technical replicates in three biologically replicated experiments.

### 4.5 Pathogenicity and infection‐related morphogenesis assays

Infection‐related appressorium development was assessed 8 hpi, following germling differentiation on hydrophobic glass cover slips (Gerhard Menzel, Glasbearbeitungswerk GmbH & Co., Braunschweig, Germany), counting 100 germlings in 3 independent experiments. Cuticle penetration was assessed, scoring frequency of penetration pegs and intracellular infection hyphae formation on onion epidermis, after incubation at 24°C for 24 hr.

Leaf infection assays were performed on blast‐susceptible, 14–21‐day‐old seedlings of rice (Oryza sativa L.) cultivar CO39 or 7‐day‐old seedlings of barley (Hordeum vulgare L.) cultivar Golden Promise, using suspension of conidia (2.5 × 10^5^/ml^−1^) in 0.2% (w/v) gelatine water, spray inoculated onto leaves as Samalova et al. (2013). For the *Δgel1Δgel3Δgel4* mutant and Guy11, healthy and abraded (with fine‐grade Emory board) rice leaves were inoculated with inverted plugs of colony edge‐growing mycelium and infection assessed 5 days later. Leaves were autoclaved in 50 ml 1 M KOH, rinsed 3× in SDW, several drops of 0.05% (w/v) aniline blue in 0.067 M K_2_HPO^4^ (pH 9.0) added and samples viewed by epifluorescence microscopy (Hood & Shew 1996).

### 4.6 Cell wall purification, fractionation and monosaccharide linkage analysis

Samples for wall analysis were prepared by scraping spores or hyphal residues (for *Δgel1Δgel3Δgel4*) from 10‐day‐old plates and inoculating 150‐ml liquid CM medium (without yeast extract), shaking at 150 rpm at 24°C for 4 or 7 days for the triple mutant. The cultures were washed three times with SDW and freeze‐dried.

Cell wall polysaccharides were purified and fractionated into alkali soluble fraction (ASF) and alkali insoluble fraction (AIF), as Mélida, Sandoval‐Sierra, Dieguez‐Uribeondo, and Bulone (2013), with three modifications: (a) mechanical disruption of mycelium with a vibratory disc mill (RS400, Retsch) in 2 cycles of 30 min at 30 Hz/s; (b) alcohol‐insoluble residue was treated with α‐amylase to remove starch/glycogen carbohydrates; (c) no SDS‐mercaptoethanol treatment.

Total carbohydrate composition analysis of the two fractions was by acid hydrolysis, derivatization of released monosaccharides to their alditol acetates, and final quantification by GC‐EI‐MS (Blakeney, Harris, Henry, & Stone 1983; Mélida et al. 2013). Mild acid hydrolysis by TFA (3 h, 121°C) was employed for ASF (Albersheim, Nevins, English, & Karr 1967); for AIF, Saeman two‐step sulfuric hydrolysis (72% H_2_SO_4,_ R.T., 3 h; diluted H_2_SO_4_, 100°C, 3 h) was applied.

Monosaccharide linkage analysis was by methylation using the CH_3_I/NaOH method (Ciucanu & Kerek 1984; Mélida et al. 2013). Partially methylated alditol acetates were analyzed by GC/EI‐MS. Monosaccharide linkages (mol%) were obtained from four technical replicates of each of three biological replicates.

### 4.7 Protoplast release by Glucanex

Three‐day‐old liquid cultures of Guy11 and *Δgel1Δgel3Δgel4* mutant, prepared as for M. oryzae transformation, were digested with Glucanex (13 mg/ml^−1^) for 60, 120, and 180 min, after which, 10‐μl aliquots were withdrawn and protoplasts counted.

### 4.8 Transmission electron microscopy

Mycelial squares (app 5 × 5 mm) were cut from the growing edge of 10‐day CM plates, fixed and viewed as described in Samalova et al. (2014).

### 4.9 Scanning electron microscopy

Guy11 and *Δgel1Δgel3Δgel4* strains were grown for 2–4 days over glass cover slips laid on CM plates and fixed in 2% aqueous osmium tetroxide for 2 h and sequentially dehydrated in ethanol/water mixtures (25, 50, 75, 95, and 100% ethanol (30 min each mixture)) and transferred to dry ethanol. Following critical point drying (Tousimis Autosamdri® 815), material was coated with gold/palladium (Polaron SC7640) and viewed in a JEOL 5510 SEM operating at 15 kV.

### 4.10 RNA seq

RNA‐seq libraries were prepared using 5 μg of total RNA isolated from 21‐day‐old cultures grown on CM plates with TruSeq SBS Kit v3 from Illumina (Agilent), according to manufacturers' instructions. One hundred base paired‐end reads were sequenced from mRNA libraries on Illumina HiSeq 2500 (Illumina, Inc.) and filtered by fastq‐mcf programme from the ea‐utils package (http://code.google.com/p/ea‐utils/), applying –x 0.01, −q 20, −p 10, and –u, and mapped to *Magnaporthe oryzae* 70–15 reference genome version 8 (Dean et al. 2005), using the TopHat2 splice site‐aware aligner (Kim et al. 2013). Counts of reads mapping to each gene in the genome were generated using the HTSeq‐count function of the HTSeq package (Anders, Pyl, & Huber 2015). Relative gene expression was quantified and differentially expressed genes identified using DESeq (Anders & Huber 2010). Gene ontology (GO) annotation of the M. oryzae genome and analysis of GO categories were performed using BLAST2GO (Conesa & Götz 2008).

## Supporting information


**Supplementary Table S1: Linkage analysis of the** ***M. oryzae*** **purified cell wall polysaccharides (mol%).**
**Supplementary Table S2: Primers for heterologous expression of *GELs* and complementation of *Δgas1*.**
**Supplementary Table S3: Primers for *GEL*s knock‐outs.**
**Supplementary Table S4: Primers for fluorescently tagged *GEL*s.**
**Supplementary Table S5: Primers for qRT‐PCR.**
**Supplementary Table S6: Up‐regulated genes in two independent *Δgel1Δgel3Δgel4* mutant strains compared to Guy11. – see file “Table_S6.xlsx”.**
**Supplementary Table S7: Down‐regulated genes in two independent *Δgel1Δgel3Δgel4* mutant strains compared to Guy11. – see file “Table_S7.xlsx”.**
**Supplementary Table S8: Up‐regulated GO_0016798 glycosyl hydrolase annotation in two independent *Δgel1Δgel3Δgel4* mutant strains. – see file “Table_S8.xlsx”.**
**Supplementary Table S9: Up‐regulated GO_0006508 proteolysis annotation in two independent *Δgel1Δgel3Δgel4* mutant strains. – see file “Table_S9.xlsx”.**
**Supplementary Figure S1: Complementation of *Δgas1* mutant in yeast.** Yeast *Δgas1* mutant, characterised by reduced growth, was complemented by yeast *GAS1* but not by M. oryzae *GEL3* or *GEL4* on galactose inducing media (SG) supplemented by Congo Red (CR), Calcofluor White (CFW) and sodium dodecyl sulphate (SDS). Wild‐type yeast (WT) transformed with *GAS1* or the pYES empty vector is shown as a control. Series of dilution at OD 0.1, 0.01 and 0.001 were used.
**Supplementary Figure S2: Plate growth and germling infection‐related development and pathogenicity assays of wild‐type, mutant and complemented** ***M. oryzae*** **strains.** Guy11, *Δgel3, Δgel4, Δgel3Δgel4* mutants, *GEL3:mCherry* and *GEL4:eGFP* complemented *Δgel3* and *Δgel4* strains, respectively; grown on **(a)** complete medium (CM), **(b)** minimum medium, **(c)** CM supplemented with CR or SDS **(d)** at 24°C for 10 days. **(e)** Development of appressoria (white) and melanised appressoria (grey) at 8 h post inoculation (hpi) of conidia onto hydrophobic surface. **(f)** Invasion hyphae formation, as elaborated from penetration pegs on onion epidermis at 24 hpi; results normalised to Guy11. **(g)** Number of lesions developed on barley (Hordeum vulgare) cultivar Golden Promise leaves spray‐inoculated with conidial suspensions (2.5 × 10^−5^ spores ml ^−1^) and incubated for 5 d for development of blast lesions; results normalised to Guy11. **(h)** Representative examples of Guy11 and the double *Δgel3Δgel4* mutant infection as described in (g) are shown. Experiments were replicated three times with two independent lines of each strain; representative pictures are shown. Results are shown as mean ± SD (e) or SEM (f, g).
**Supplementary Figure S3: qRT‐PCR results of *GEL* expression during spore development, early infection and in mycelia.**
**Supplementary Figure S4: Cryo‐SEM images of *Δgel1Δgel3Δgel4* mutant and Guy11.** 2 mm from the growing mycelial edge at 1.000× (left) and 120× (middle and right) magnification.
**Supplementary Figure S5: RNA‐seq analysis of *Δgel1Δgel3Δgel4* mutant strains and wild‐type Guy11. (a)** Heatmap showing Euclidean distance between replicates of each sample (Guy11 and two independent *Δgel1Δgel3Δgel4* triple mutants) as calculated from variance‐stabilising transformation of the count data. **(b)** GO terms over‐represented in genes significantly up‐regulated and **(c)** down‐regulated (when compared to Guy11: adjusted *P*‐value <0.01, moderated log_2_‐fold change expression >1) in both triple mutants (Test Set) as compared to all genes in the genome (Reference Set) (using Fisher's Exact Test, FDR < =0.05).
**Supplementary Figure S6: Southern blot analysis of all mutant strains.**
**Supplementary Figure S7: Schematic pictures of complementation constructs and list of strains.**
**Supplementary Table S1: Linkage analysis of the** ***M. oryzae*** **purified cell wall polysaccharides (mol%).**
**Supplementary Table S2: Primers for heterologous expression of *GELs* and complementation of *Δgas1*.**
**Supplementary Table S3: Primers for *GEL*s knock‐outs.**
**Supplementary Table S4: Primers for fluorescently tagged *GEL*s.**
**Supplementary Table S5: Primers for qRT‐PCR.**

Supporting info item
